# Transcriptomic and metabolomic insights into light-mediated unicellular-to-multicellular transition in *Dictyostelium discoideum*

**DOI:** 10.1098/rsob.250125

**Published:** 2025-10-08

**Authors:** Yuehui Tian, Huiru Liu, Shanshan Xu, Zihe Wang, Zhili He, Ruiqi Liu, Longfei Shu

**Affiliations:** ^1^ School of Life Sciences, Guangzhou University, Guangzhou, People’s Republic of China; ^2^ Environmental Science, Sun Yat-Sen University School of Environmental Science and Engineering, Guangzhou, People’s Republic of China; ^3^ Southern Marine Science and Engineering Guangdong Laboratory–Zhuhai, Zhuhai, Guangdong, People’s Republic of China; ^4^ School of Marine Science, Sun Yat-sen University–Zhuhai Campus, Zhuhai, Guangdong, People’s Republic of China

**Keywords:** *Dictyostelium discoideum*, light-induced cell development, unicellular, multicellular

## Abstract

*Dictyostelium discoideum* is a social amoeba that transitions from unicellular to multicellular forms in response to environmental signals, making it an intriguing model for studying cell aggregation and differentiation. Although previous studies have demonstrated that *Dictyostelium* slugs exhibit phototaxis, the mechanisms behind light-induced developmental changes remain unclear. In this study, we investigated how light triggers the transition to multicellularity and its associated metabolites and genes. Our findings revealed that spore yield depends on light exposure, with slower multicellular development under dark incubation. Transcriptomics analysis on QS9 amoebae identified upregulation of small GTPases such as *rasD* and *racL* in response to light, which likely promote cell movement, phagocytosis and actin protrusions. Light also enhanced cAMP production, driving the aggregation, post-aggregation and development of single cells. Additionally, c-di-GMP was essential for cell differentiation during multicellular growth and was upregulated by light. Metabolomic analysis on QS9 amoebae revealed that the downregulation of LPC (lysophosphatidylcholine) was detected under both unicellular and multicellular phases. Moreover, reduced levels of GSH (glutathione) in dark may impede multicellular structures of *D. discoideum*. These findings provide insights into light-triggered cell differentiation and pattern formation, offering a better understanding of molecular mechanisms underlying the transition to multicellularity in *Dictyostelium* cells.

**Figure:**
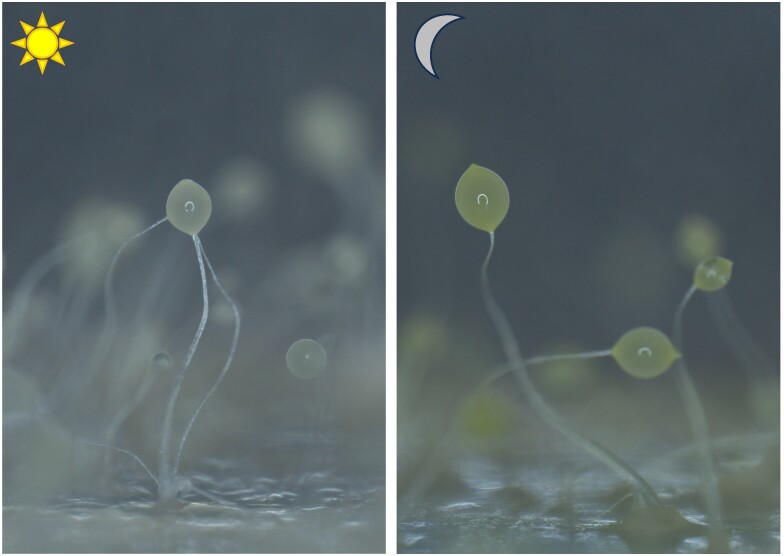

## 1. Introduction

Dissecting the molecular mechanisms underlying the transition from unicellular to multicellular cell development is a fundamental task of evolutionary biology. Investigating conserved core signalling genes or metabolites involved in the development of both unicellular and multicellular cells would allow us to understand the mechanisms that regulate this critical transition. *Dictyostelium discoideum* contains different cell types during the life cycle, including solitary vegetative cells germinated from spores, multicellular slugs and fruiting bodies with new spores [1]. Therefore, using *Dictyostelium* to study this transition can help elucidate the underlying molecular mechanisms driving changes from unicellular to multicellular phases.

Several genetic modules, including guanylyl cyclase, cGMP-binding protein C and phosphatidylinositol-3-OH kinases (PI3Ks), have effects on the direction of migration by electrotaxis in *Dictyostelium* cells. It was reported that approximately 55 genes are involved in phototaxis during the multicellular slug stage, including several of the encoded proteins regulating signal transduction pathways related to the intracellular messengers such as cAMP, cGMP, inositol 1,4,5 trisphosphate and Ca^2+^ [2,3]. As second messengers, cyclic nucleotides like cAMP and cGMP exert distinct effects on various signal transduction processes in *Dictyostelium discoideum*. During the starvation phase, amoeba single cells can secrete cAMP to mediate chemotaxis for cell aggregation, which in turn regulates gene expression throughout development [4]. By responding to the extracellular concentration of cAMP with synchronized oscillation pattern, individual amoeba cells can transition from quiescence to rhythmic activity of collective behaviour known as ‘quorum sensing’ [5]. It is possible to unveil the evolutionary role of cAMP in the morphogenesis and differentiation of *D. discoideum* through a comparative analysis of cAMP signalling in social amoebae during stress responses at the unicellular stage [6]. In addition, cGMP signalling is involved in the regulation of the rear of the cell during the aggregation stage, maintaining stability of head-to-tail cell contacts [7].

In the multicellular stage of *Dictyostelium*, other signalling molecules have essential effects on the cell differentiation and development. As a second messenger, c-di-GMP is known to induce biofilm formation in prokaryotes, its role in extracellular matrix deposition during *Dictyostelium* development has been investigated. In the slug stage of *Dictyostelium*, diguanylate cyclase (DgcA) can synthesize c-di-GMP, which promotes stalk cell differentiation and fruiting body formation [8,9]. Ras small-GTPases are vital in mediating chemotaxis in response to cAMP and folic acid gradients within *Dictyostelium* cells [10]. A general model suggests that that small GTPases can activate spatial signals that integrate to form phagocytic cups for engulfing various targets [11]. A phototaxis signalling complex has been identified through protein coimmunoprecipitation, including proteins such as RasD, filamin, ErkB, GRP125 and PKB [12]. Based on the mutants lacking RasD and several RasGEFs suggested that the Ras signalling pathway is involved in the phototaxis [13]. However, it remains unclear whether both unicellular and multicellular stages utilize a similar core signalling complex. In *Dictyostelium* cells, dDia1 is co-localized with filamentous actin throughout the entire pseudopod. A knockout mutant dDia1 showed a markedly impaired phototaxis as well as smaller fruiting bodies during the multicellular stage [14].

Chemotaxis is vital for the migration of protists, but the underlying molecular signals involved remain poorly understood due to the complex metabolites produced by diverse bacteria in the environment. The interaction between host *Dictyostelium* and its symbiont *Burkholderia* is chemotaxis-dependent [15,16]. *D. discoideum* can selectively detect and feed on various soil bacteria [17]. The establishment of amoeba–bacterium symbiosis may rely on the symbiont-induced phagosome biogenesis [18]. However, it is still challenging to identify specific bacterial metabolites as chemoattractants for the amoeba migration and amoeba–bacterium symbiosis [19]. In *Dictyostelium* cells, chemoattractant gradients are sensed by G-protein coupled receptors (GPCRs), which regulates downstream signalling events [20]. Similarly, in human cells like neutrophils, GPCR-mediated chemotaxis in response to chemoattractant is evolutionarily conserved similar with *Dictyostelium* cells [21]. It was reported that a metabolite lysophosphatidylcholine (LPC) can activate various downstream MAPK signalling pathways by binding to GPCRs and Toll-like receptors, thus inducing the chemotaxis or migration of lymphocytes and macrophages [22]. However, it remains unclear whether environmental factors, such as light exposure, can have effects on the chemotaxis of *Dictyostelium* cells.

Some photosynthetic microorganisms like green algae and cyanobacteria are capable of production and development with photosystems. Other non-photosynthetic microorganisms like fungi and protists are still capable of responding to light because of their photoreceptors. Photoreceptors such as Cyclops (cyclase opsins), bPAC (photoactivated adenylyl cyclase) or phytochrome exist in green algae, fungi, protists and certain bacteria. Those photoreceptors can absorb light of different wavelengths from blue light to far-red light, thus stimulating physiological processes including phototaxis in *Chlamydomonas* and *Blastocladiella* [23–28]. A multicellular *Choanoeca flexa* was identified and described with a light-regulated colony inversion, which can be regulated by a membrane rhodopsin phosphodiesterase (RhoPDE) [29]. These observations allow us to understand how light stimulation could induce the development of differentiation processes of amoebae. It is obvious that the migration of amoeba slugs are phototaxis, but the molecular mechanisms or photoreceptors behind still need to be clarified. It was reported that the synthesis of pigment carotenoids reached a peak in the fruiting body stage of *D. discoideum*, which sharply declined in the vegetative stage [30]. We observed the accumulation of yellow coloration in the fruiting body under dark, which can be bleached upon exposure to light. We propose that the colour change is primarily due to the bleaching effect of pigments.

In this study, we explored the molecular mechanisms underlying the transition from unicellular to multicellular stages of *Dictyostelium* cells in response to dark and light treatment. Notably, the morphogenesis differs significantly in multicellular slugs. We observed that phototaxis of slugs is markedly enhanced under light stimulation compared with darkness. Our findings indicate that light exposure can improve and accelerate the transition from unicellular to multicellular forms, resulting in a higher yield of germinated spores in fruiting body. Transcriptomic analysis indicates that upregulated genes such as multiple small GTPases and membrane bound GPCR may play a role in mediating light responses and promoting cell proliferation. In the unicellular stage, some downregulated genes including adenylate cyclase (*acrA*) and cyclic AMP receptor (*CarB*) could reduce the cell aggregation during dark incubation. Furthermore, we also observed some metabolites including lysophosphatidylcholine (LPC) were downregulated under dark treatment; however, whether LPC may negatively impact migration in the unicellular and multicellular stages of amoeba cells is still unclear. Overall, this study suggests that conserved molecular mechanisms may influence the transition from the unicellularity to multicellularity, and that light exposure, as one of key environmental factors, may facilitate and enhance this transition towards multicellularity.

## 2. Material and methods

### 2.1. Culture conditions of amoeba clones

Amoebae were cultured on SM/5 agar plates by mixing 2 × 10^5^ *D. discoideum* spores with the food-source bacteria *K. pneumoniae* (OD_600_ = 1.5, 200 μl). To dilute and transfer amoeba spores or bacteria, KK2 buffer was prepared with 2.25 g KH_2_PO_4_ (Sigma-Aldrich) and 0.67 g K_2_HPO_4_ (Sigma-Aldrich) per litre. The ingredients of SM/5 agar plates contain 2.0 g BactoPeptone (Oxoid), 2.0 g of yeast extract (Oxoid), 1.9 g KH_2_PO_4_, 1.0 g K_2_HPO_4_, 2.0 g glucose (Tianjin Damao), 0.2 g MgSO_4_ (Tianjin Damao) and 15.0 g agar (Oxoid) per litre. In this study, we selected three *D. discoideum* isolates (NC4, QS9, QS9 with symbiotic B1QS70) to examine their photosensitivity (i.e. how the dark and light influence their development). In nature, *D. discoideum* NC4 and QS9 have no symbiotic bacteria. Here we mixed QS9 with symbiotic *Paraburkholderia agricolaris* B1QS70 to artificially induce the symbiosis of QS9 with B1QS70. *P. agricolaris* B1QS70 was also cultured on SM/5 agar plates and the QS9-B1QS70 symbiosis was established by mixing 10 μl (OD_600_ = 1.5) B1QS70 with amoeba spores and *K. pneumoniae* as above on each plate. All plates were cultured at 21℃. Control groups were cultured in an incubator with normal white light illumination. Other samples were cultured either in incubators without light (dark treatment group) or incubators illuminated by light treatment of different wavelengths (blue at approx. 450 nm, green at approx. 530 nm and red at approx. 650 nm with the same intensity at approx. 2 mW cm^−2^). Light intensities were measured with a fibre optic spectrometer (FLA5000, Hangzhou Flight Technology Co.). In each experiment, the same batch of amoeba spores or bacteria was employed. Under treatment with various concentrations of diphenylamine (DPA) (Shanghai Macklin), we first prepared a 100 mM stock solution of DPA dissolved in 95% ethanol. Subsequently, the stock solution was added to the culture media to achieve final concentrations of 10 μM, 20 μM and 50 μM. Control groups were prepared with an equivalent volume of ethanol in the culture media.

### 2.2. Spore yield assays

Under light of different wavelengths, white light or dark conditions, we only used 2 × 10^5^ *D. discoideum* spores QS9 mixed with 200 μl *K*. *pneumoniae* (OD_600_ = 1.5) for comparing total spore numbers on each SM/5 agar plate. The incubation time was normally designed for 5−6 days after plating. Three *D. discoideum* clones including QS9, QS11 and QS70 were selected to test spore yield under different light illumination time. To harvest spores, 5 ml KK2 buffer with 0.1% Nonidet P-40 was prepared to clean out the fruiting body on agar plate. The collected spores from above fruiting body were vortexed and diluted 10 times. Then 10 μl was aspirated to a haemocytometer to count the spore numbers by using a light microscope with 40× objective lens and then the total number of spores were individually calculated in each plate. No less than three biological replicates were employed in each group under dark and other light wavelengths treatment as indicated in each figure legend.

### 2.3. Amoeba slug migration assays

To test the migration rate, we chose to use the multicellular slugs in comparison between dark and light treatment. Before the incubation, 2 × 10^5^ *D. discoideum* QS9 spores was mixed with feeding bacteria *K. pneumoniae* (OD_600_ = 1.5, 200 μl). Then the mixture sample was diluted five times, and 5 μl diluted sample were separately loaded on each SM/5 agar plate. The plates were covered by foil paper to mimic dark treatment, while other plates were covered by foil with one side open to mimic the light treatment. Then we chose to select slugs in the front end of plates and take two time points to measure the migration distance (cm) from the beginning loading point, including 48 h and 72 h during the migration of slugs. The migration rates (cm per day) were finally calculated.

### 2.4. Microscopy and phenotyping analyses

In the unicellular stage, amoeba vegetative cells under both dark or light conditions were taken after 36 h incubation with feeding bacteria *K. pneumoniae*. The images in bright field were taken by a Zeiss LSM 980 laser scanning microscope with 40× objective lens (400× total magnification). In the multicellular stage, amoeba slugs at 48 h and fruiting bodies at 5 days were selected for imaging. Images of multicellular amoebae under different light wavelengths were captured by a Zeiss Axio Zoom.V16 stereo microscope. In comparison of the photosensitivity of amoebae at different light illumination time, images of the fruiting bodies on the plates were acquired by using a digital camera (EOS M6, Canon).

### 2.5. Statistical analysis

We chose to use the GraphPad Prism 10 software package to do statistical analyses. All the spore yield assays were performed at least three biological replicates as indicated in figure legends. In the figures, data are shown with individual points, mean values and standard deviations (SD). Under light and dark treatment, different spore yield assays were analysed by employing an unpaired two-tailed Student’s *t*-test. In comparison among different amoeba clones QS9, QS11 or QS70 under light and dark treatment, multiple *t*‐test was used among individual groups. For multiple comparison among different light wavelengths or light illumination time, statistical analyses were performed by employing ANOVA with Tukey’s multiple-comparison test. *p*-values and statistical significance were determined as indicated in figure legends (* indicates *p* < 0.05; ** indicates *p* < 0.01; *** indicates *p* < 0.001; and **** indicates *p* < 0.0001).

### 2.6. Transcriptomic and metabolomic analysis

In the unicellular and multicellular stages, the amoeba cells were selected for both transcriptomic and metabolomic analysis in parallel. Under constant dark and light treatment, amoeba QS9 vegetative cells (approx. 1 × 10^7^) were harvested at 36 h, respectively. During the multicellularity, amoeba QS9 slugs were harvested at 48 h in both dark and light treatment. During the collection processes, we used approximately 2 ml ice precooling KK2 buffer to wash out the mixture with amoeba cells and feeding bacteria from agar plates. Then the mixture was centrifuged (1000 × g, 4℃ for 3 min) to remove extracellular bacteria from the supernatant. The extracellular bacteria were removed by the above washing steps with three times centrifugation. The light treatment was designed as the control group while the dark treatment was regarded as the treatment group with three biological replications in each condition.

For the transcriptomic analysis, the cell collection processes were operated on ice and 1 ml TRIzol reagent (Invitrogen, Carlsad, CA, USA) was used to resuspend cell pellets in each individual experiment. After RNA extraction, the RNA quantity and quality were evaluated to ensure suitability for establishing sequencing libraries, which were generated by using NEBNext UltraTM RNA Library Prep Kit for Illumina (NEB, USA) at Metware Biotechnology Co. (Wuhan, China). Oligo(dT) beads were used to enrich eukaryotic mRNA with poly-A tails, which inherently reduced the likelihood of bacterial RNA included in the sequencing reads. Fastp v 0.19.3 was used to filter the original data and create clean reads, which were used for all subsequent analyses. *D. discoideum* reference genome (GCF_000004695.1) was used to construct the index and compare with clean reads by HISATv2.1.0. The mapped reads accounted for approximately 90% in all samples, indicating that the bacterial contaminants were filtered out without influencing the subsequent transcriptomic analysis. RSEM Version 1.3.3 was used to calculate the gene alignment with transcripts per kilobase million (TPM) as original quantitative results. Differentially expressed genes (DEGs) were identified between the control and treatment groups in each developmental stage by using DESeq2 v1.22.1. Significant expressed genes were adjusted to satisfy log2 fold change > 1 and *p*‐value < 0.05. The volcano plots were drawn to show DEGs with *x*-axis log2 (S1/S2, S2 indicates the control group). Gene ontology (GO) enrichment was analysed by Goatools in the DEGs. The corrected *p*-value less than 0.05 was chose in GO terms regarded as significant enrichment.

In terms of metabolomic analysis, the supernatant from the same batch of samples were extracted by adding 500 μl 80% methanol-water internal standard extractant with vortex and centrifugation steps. All samples were analysed by LC-MS system by the Metware Biotechnology Co. (Wuhan, China). In comparison between dark treatment and light control groups, significantly regulated metabolites were determined by variable importance in projection VIP>=1. VIP values were obtained from orthogonal partial least-squares differentiation analysis (OPLS-DA) model. Differentially accumulated metabolites (DAMs) shown in volcanic plot were screened by the absolute log2 fold change ≥ 1 and *p*-value < 0.05. The top 20 DAMs (names of compounds) were shown with bar chart in positive ion mode, while all the DAMs were shown in negative ion mode. Kyoto Encyclopedia of Genes and Genomes (KEGG) compound database was used to annotate the functions of DAMs.

## 3. Results

### 3.1. The growth condition of *Dictyostelium* cells is dependent on light exposure

**Figure 1.**
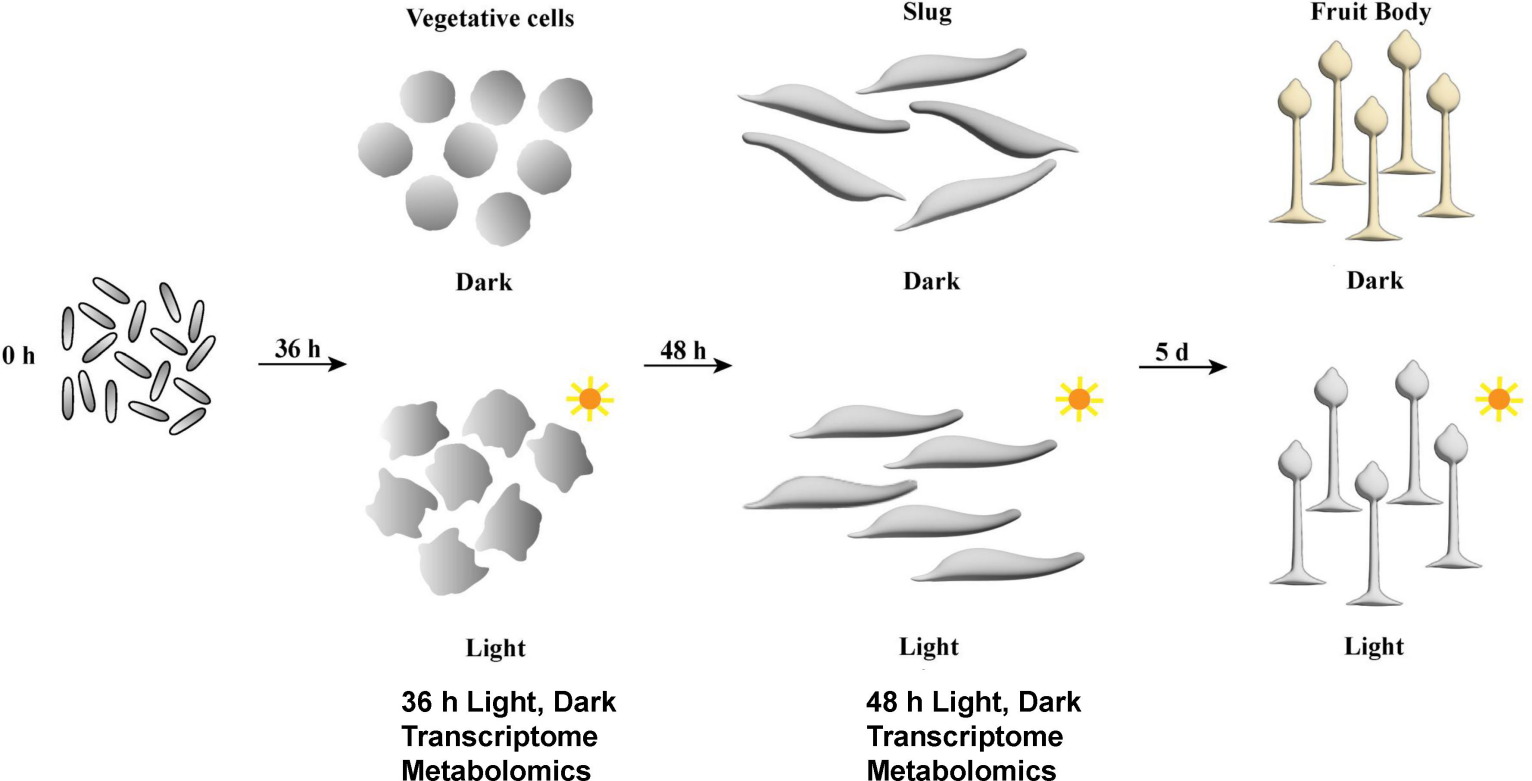
Schematic model of the transition from unicellular to multicellular development of amoebae incubated under light and dark conditions. Amoeba spores were initially mixed with feeding bacteria. In the unicellular stage, vegetative cells germinated from spores after 36 h of incubation under both dark and light conditions. In the multicellular stage, slugs were formed after 48 h of incubation. After 5 days, differences in the coloration of fruiting bodies were shown when comparing dark and light conditions. Vegetative cells at 36 h and slugs at 48 h were collected for transcriptomic and metabolomic analyses.

Throughout the life cycle of *Dictyostelium* cells, distinct morphological differences from unicellular to multicellular stages can be clearly recognized. Here we mainly focused on investigation of three representative stages during the development of *Dictyostelium* cells, including unicellular vegetative cells after 36 h of incubation, the multicellular slugs after 48 h, and the fruiting body stage after 96 h (figure 1). We compared the morphologies of *Dictyostelium* cells at these three developmental stages under both dark and light conditions. To illuminate the molecular mechanisms underlying the transition from unicellular to multicellular stages under different light conditions, we analysed the transcriptome and metabolomics of vegetative cells at 36 h and multicellular slugs at 48 h, as they remain similar cell types in either developmental stage (figure 1).

**Figure 2.**
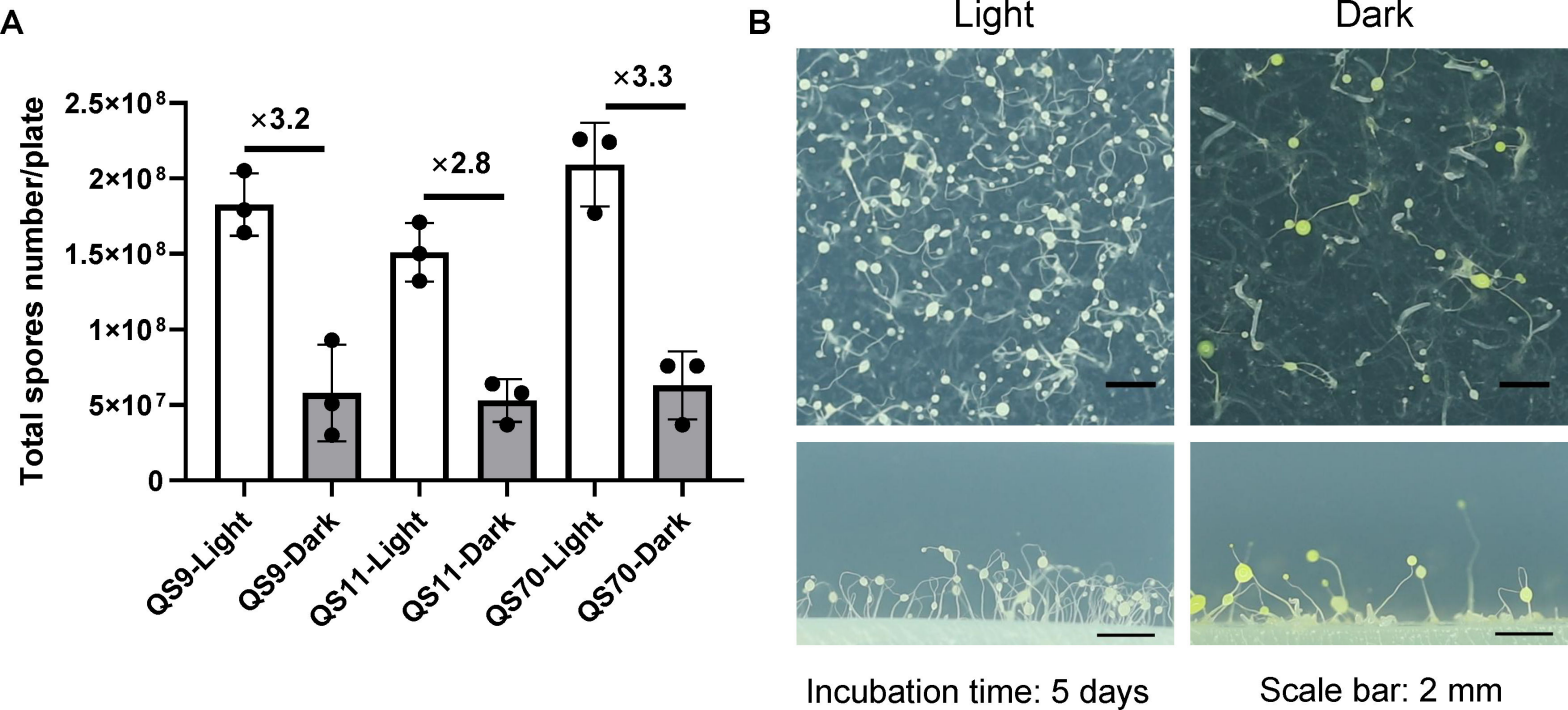
Reduction in the total number of amoeba spores and multicellular development under dark incubation. (A) Comparison of total number of spores on each plate under light and dark incubation for amoebae QS9, QS11 and QS70. Three biological replications were performed under both dark and light conditions (data are presented as mean ± SD of three independent experiments). (B) Differences in developmental morphology between light and dark treatments after 5 days of incubation (scale bar = 2 mm).

When comparing the morphological differences in the fruiting body after 5 days incubation, we first observed that the number of *Dictyostelium* spores on each plate was dramatically increased under light illumination, about three times higher than that of in the dark (figure 2A). This light-enhanced spore yield in fruiting body was detected in different amoeba clones, including QS9, QS11 and QS70. We further imaged the fruiting body on SM/5 agar plates, and the number of fruiting bodies is obviously higher under light illumination than that of in the dark (figure 2B). The development of fruiting bodies in the dark appeared delayed and incomplete compared with those cultivated in the light. Furthermore, the yellow coloration of fruiting bodies and the growth medium obviously appeared under dark treatment, while the fruiting bodies exposed to light incubation remained white (figure 2B). This observation suggests that certain metabolites could be produced or accumulated in the absence of light.

### 3.2. The coloration and spore number of *D. discoideum* is sensitive and switchable in response to light

As illustrated in figure 2, we observed different morphologies of fruiting bodies under dark and light conditions. Here we verified that the morphology of fruiting bodies of *Dictyostelium* cells can be recovered from dark to light exposure. To demonstrate this phenomenon, we utilized three different amoeba clones NC4, QS9 and QS9 with symbiotic *Paraburkholderia agricolaris*, all of which exhibit similar photo-switchable phenotypes. When the three amoeba clones were incubated under constant dark or light for 5 days, it is clear to see the yellow-coloured fruiting bodies in the dark and the white-coloured fruiting bodies with light illumination (electronic supplementary material, figure S1). If the amoeba cells were transferred to light exposure after 4 days of incubation in the dark, the yellow coloration of fruiting bodies then changed to white colour only after 1 day of light exposure. Our data also indicate that photo-bleaching effects can arise in the different strains, including symbiotic amoebae. This observation suggests that certain metabolites may accumulate under dark, with their levels subsequently decreasing after light exposure. These metabolites are probably pigments exhibiting light-bleaching effects. As reported previously, carotenoids were recognized as pigments that accumulated during the fruiting body stage [30]. To investigate this further, we assessed whether the deep yellow coloration could be bleached by adding different concentrations of diphenylamine (DPA) to the culture media. Under dark treatment, the bleaching effect on the fruiting bodies was not fully realized with a 10 μM DPA supplement, which resulted in a light yellow coloration (electronic supplementary material, figure S2A). However, we observed that the accumulated deep yellow coloration of fruiting bodies could be effectively bleached with 20 μM and 50 μM DPA in the culture media (electronic supplementary material, figure S2B,C). This suggests that the accumulated yellow pigments are predominantly carotenoids in dark. It is also evident that the fruiting bodies under the treatment of DPA became less and smaller compared with control groups, particularly at concentrations of 20 μM and 50 μM (electronic supplementary material, figure S2). Nonetheless, it remains unclear whether the accumulation of carotenoids or other metabolites under dark have effects on the development of *Dictyostelium* cells. Therefore, the metabolomic analysis is worth investigating to identify potential metabolites involved in the light-regulated developmental processes that occur during the multicellular fruiting body stage.

We also examined the morphology of fruiting bodies at different light exposure time within 5 days of incubation, noting that the number of new spores produced in fruiting bodies is largely dependent on light illumination time. In amoeba QS9, we observed that the number of productive spores was increasing as the exposure time was extended from 36 h to 72 h, with the total number of spores after 72 h of light incubation being equivalent to that after full light exposure for 5 days (electronic supplementary material, figure S3A). Although the other two amoeba clones QS11 and QS70 also exhibited an increasing trend in spore yield under light illumination, the number of spores produced after 72 h of light was still lower than that achieved under full light exposure time (electronic supplementary material, figure S3B,C). This indicates that the amoeba QS9 is more sensitive to light compared with the other two amoebae QS11 and QS70; however, the light stimulation remains crucial for the yield of new spores.

**Figure 3.**
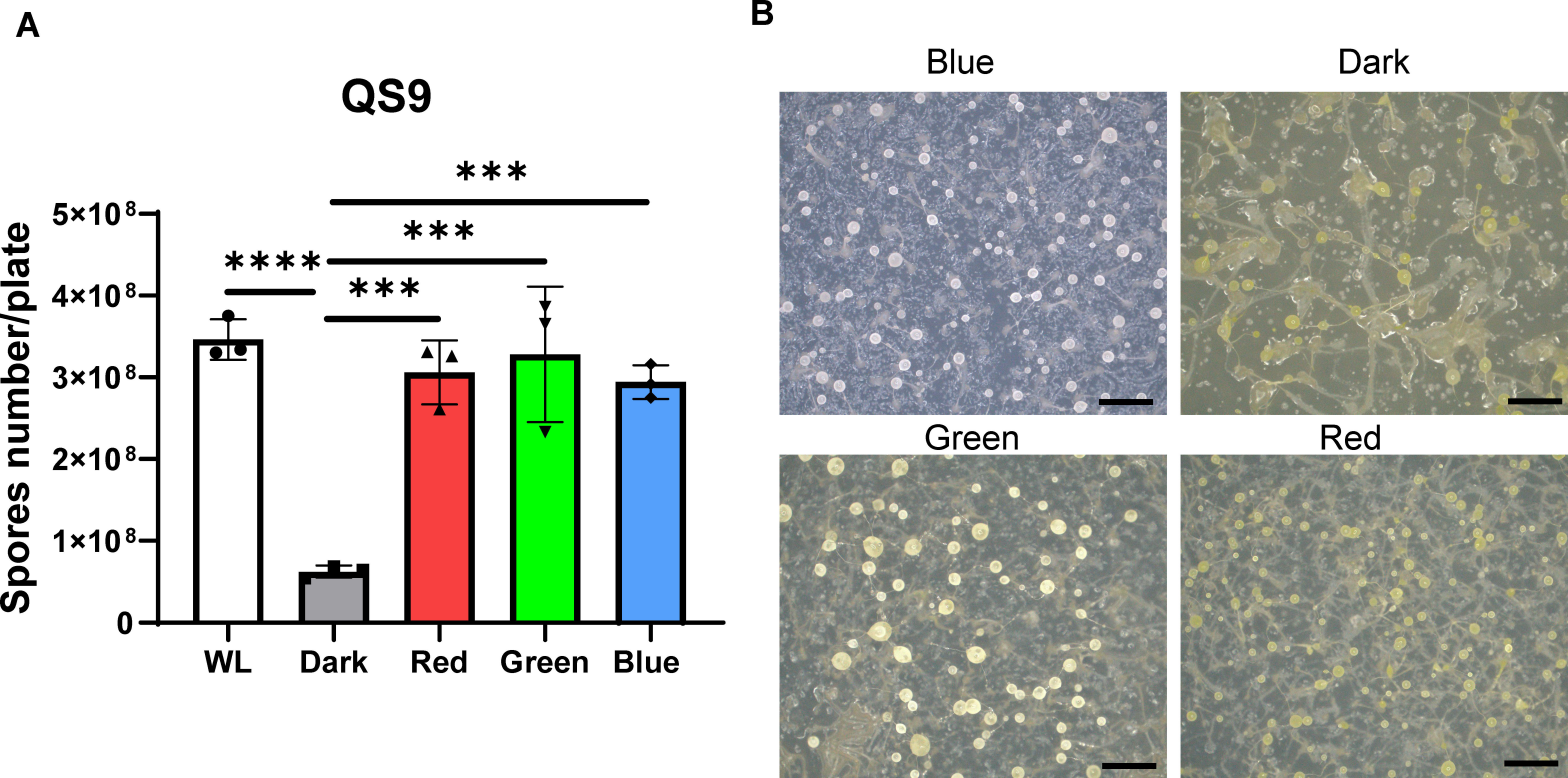
The yield of spores under different light wavelengths. (A) Comparison of total number of spores (amoeba QS9) on each plate under white light, dark and three different light wavelengths (red at approx. 660 nm, green at approx. 530 nm and blue at approx. 450 nm). Three biological replications were performed under each light condition (data are presented as mean ± SD). Each independent experiment with varying light wavelengths was analysed by using ANOVA with Tukey’s multiple-comparison test (***p < 0.001, ****p < 0.0001). (B) The different colorations of fruiting bodies (amoeba QS9) on each plate are shown for the dark condition and three light wavelengths (scale bar = 2 mm).

Additionally, we investigated the effects of different light wavelengths (blue at approx. 450 nm, green at approx. 530 nm and red at approx. 650 nm) at the same light intensity of approximately 2 mW cm^−2^ on the morphology and spore yield of *Dictyostelium discoideum*. Our results indicated that spore yield is not dependent on the light wavelength, as the number of spores incubated under blue light has no significant difference compared with that of under red and green light. Moreover, spore yield in white light is still similar with that of under different wavelengths (figure 3A). This suggests that amoeba spore yield cannot be influenced by light wavelengths. The specific membrane-bound rhodopsin or cytosolic photoreceptor involved in this sensitivity remains to be identified. In terms of morphology, we observed the fruiting bodies exhibited a white coloration under blue light exposure, similar to those under white light (figure 3B). However, the deep-yellow pigment of fruiting bodies was observed in red light incubations similar with that of under dark. The light-yellow coloration of fruiting bodies was observed in green light (figure 3B). This indicates that the colour change is likely to be due to the pigment bleaching effects, which can be regulated by different light wavelengths with absorption spectrum peaking in the blue light range.

### 3.3. Characterization of the development of *Dictyostelium* cells from unicellular to multicellular stages in dark and light

**Figure 4.**
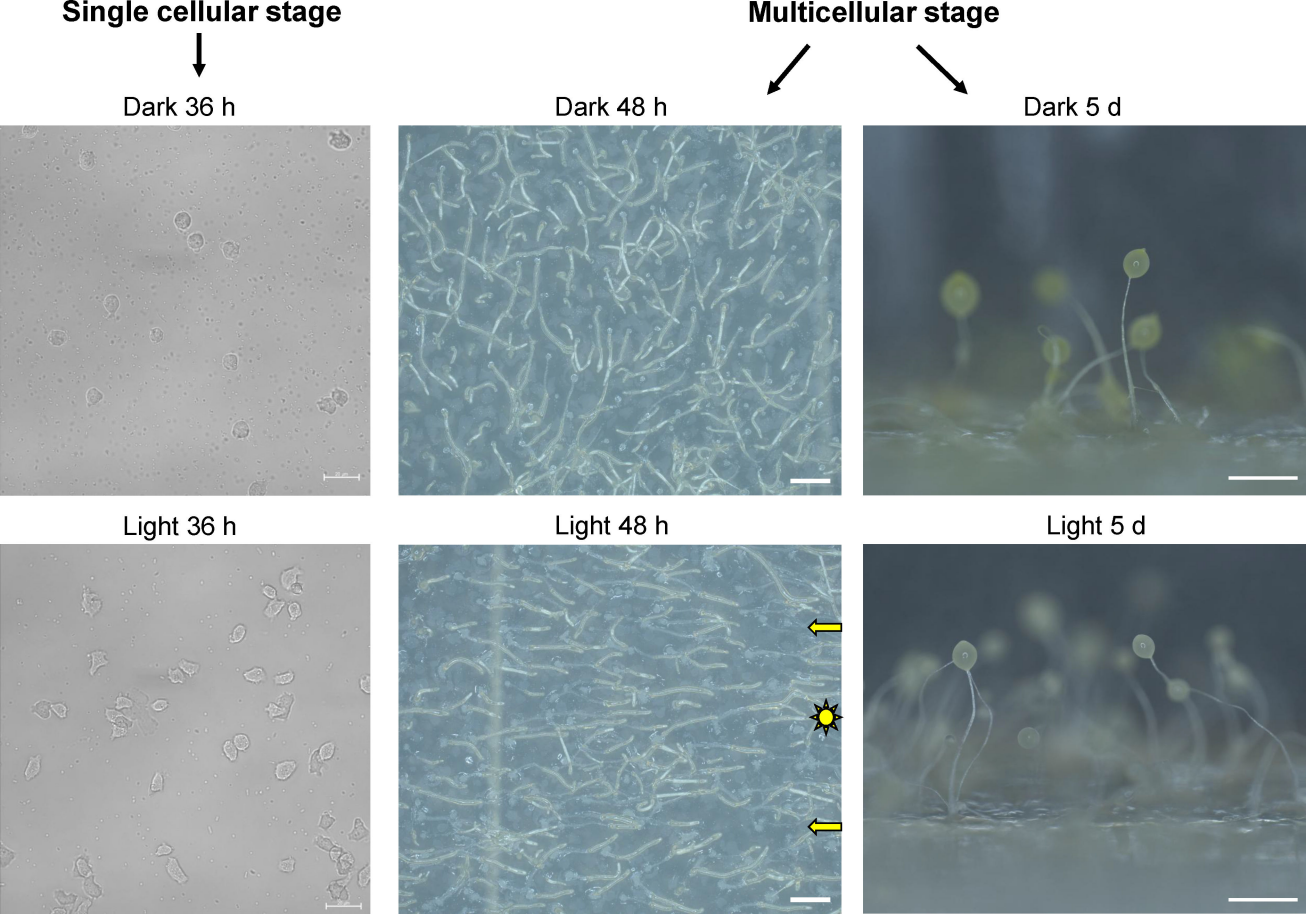
Developmental morphologies from unicellular to multicellular stage under dark and light incubations. Images of unicellular stage were taken at 36 h (scale bar = 20 μm) under both dark and light incubations. Images of multicellular slugs and fruiting bodies (amoeba QS9) were captured at 48 h and 5 days of incubation, respectively (scale bar = 1 mm). For the slugs, light illumination was directed from one side during incubation. Three biological tests showed similar morphologies under both incubation conditions.

To further investigate the morphological changes of *Dictyostelium* cells that transition from unicellular to multicellular stages, we chose to use amoeba QS9, which showed highest sensitivity to light among the examined clones. During the unicellular stage, we examined vegetative cells that germinated from amoeba spores after 36 h of incubation. Images were shown that the majority of vegetative cells maintained a rounded shape in the dark, whereas those grown under light exposure exhibited diverse shapes, primarily due to the early formation of pseudopod of amoeba cells (figure 4). In the multicellular slugs after 48 h of incubation, we can clearly see the slugs in dark forming chaotic migration directions, while the slugs can migrate to light in direction. Here we quantified the migration rates of slugs between dark and light treatment. Amoeba spores were mixed with its food bacteria and dripped on agar plates, and then the migration distances were imaged and measured at 2 days and 3 days respectively in both dark and light incubation. The data showed that the migration rate (calculated as migration length per day) in light was approximately twice that observed in darkness, when the slugs at forefront end were selected for the calculation (electronic supplementary material, figure S4). Moreover, there were significantly more slugs leading the migration in light compared with those in the dark. In terms of fruiting body formation, variations in morphology and colour were noted as previously descripted. Different cell types co-exist during the fruiting body stage including new spores and stalk cells, which complicates transcriptomic and metabolomics analyses. Therefore, here we chose to use the unicellular vegetative cells at 36 h and multicellular slugs at 48 h with similar cell types for our subsequent transcriptomic and metabolomic analyses. This approach enables us to better understand the conserved molecular mechanisms underlying the transition to multicellularity, particularly in relation to light conditions.

### 3.4. Transcriptomic analysis reveals conserved signalling molecules involved in the light-induced development of amoebae

**Figure 5.**
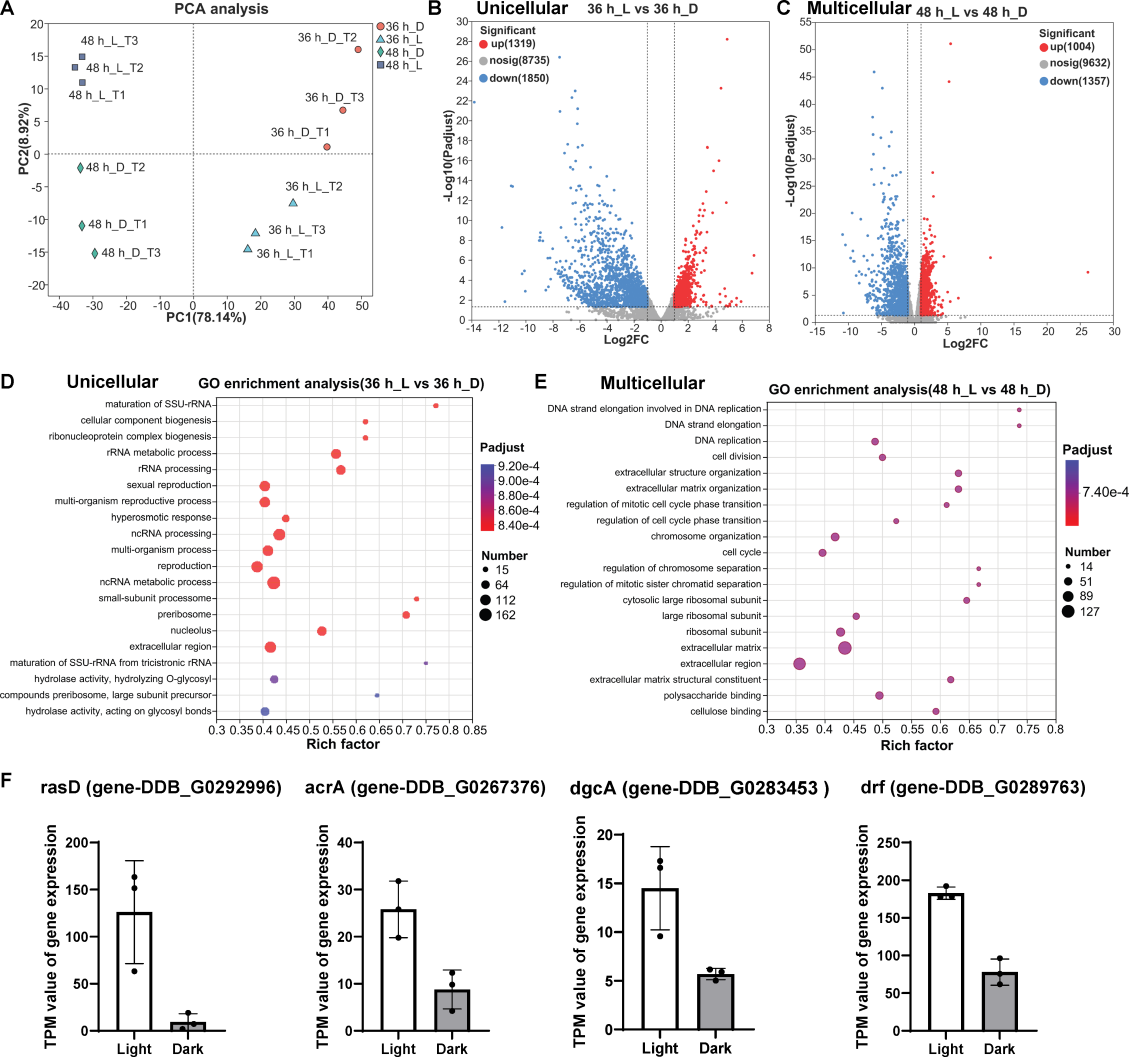
Transcriptomic analysis of unicellular and multicellular phase under light and dark treatments. Three biological replications (amoeba QS9) were performed with dark as the treatment group and light incubation as the control group. (A) PCA analysis of the four different groups. (B) Volcanic plot of DEGs in unicellular stage. (C) Volcanic plot of DEGs in multicellular stage. (D,E) The GO enrichment analysis of DEGs in the respective unicellular or multicellular stages. (F) The TPM levels of typical genes related to the development of unicellular (for rasD and acrA) and multicellular stages (for dgcA and drf).

Given the distinct morphologies at different developmental stages, here we chose to use the unicellular cells at 36 h incubation and multicellular slugs at 48 h incubation for a comparative analysis of gene expression patterns under both dark and light conditions. Based on principal component analysis (PCA), the expressed genes in unicellular and multicellular were clearly separated with PC1 at 78.14%. Similarly, the expressed genes under dark and light incubation at each incubation time (36 h or 48 h) were also obviously separated with PC2 at 8.92% (figure 5A). This clustering of three biological replicates under each treatment suggests that the transcriptomic data is reliable for further investigation. In this study, the control group is amoeba cells under light incubation, and the treatment group is amoeba cells with dark condition. Differentially expressed genes (DEGs) were selected based on over twofold change with *p*-adjust value less than 0.05. In the unicellular stage (36 h), 1319 genes (11% of the total genes) were upregulated, while 1850 genes (16% of the total genes) were downregulated under dark treatment (figure 5B). In the multicellular stage (48 h), 1004 genes (8% of the total genes) were upregulated and 1357 genes (11% of the total genes) were downregulated under dark incubation (figure 5C).

The GO enrichment analysis showed that DEGs in dark or light during amoeba development have essential effects on the regulation of various biological processes. Here we chose to show the TOP20 enriched GO terms associated with significant DEGs (figure 5D,E). In the unicellular status, DEGs were identified and involved in some biological processes (figure 5D), including rRNA and ncRNA metabolic processes, multi-organism process and reproduction, etc. Notably, several Rho GTPase and Ras GTPase genes were downregulated under dark treatment, which play essential roles in regulating biological functions such as phagocytosis, engulfment as well as chemotaxis. Therefore, we suggested that the light exposure may enhance those biological functions as descripted above. We also identified specific genes related to the phototaxis or chemotaxis that were downregulated under dark treatment. For example, *rasD* gene (Ras GTPase RasD) is downregulated about 13 times under dark incubation compared with its expression level with light exposure in the unicellular phase (figure 5F). *FLN* was previously reported to involve in development and phototaxis of amoebae, which was also downregulated about threefold under dark (electronic supplementary material, figure S5A). As a crucial chemotaxis molecule, cyclic adenosine monophosphate (cAMP) can be secreted in the unicellular cells and plays key roles in regulating cell aggregation during the starvation. Here we also observed that the adenylate cyclase gene *acrA* was downregulated under dark in the unicellular phase. This indicates that the light exposure can enhance the production of cAMP, thereby improving the cell aggregation (figure 5F). Furthermore, in the multicellular stage, DEGs were identified and involved in additional biological processes (figure 5E), including extracellular matrix, cell division as well as cell cycle, etc.

Many cellulose-binding domain-containing genes such as *staA*, *ecmF* can improve the multicellular development and fruiting body formation, which were significantly downregulated under dark incubation. This also provides insights that dark incubation can impede the multicellular development as we previously observed. We also found that a weak actin nucleator named *drf* ( = diaphanous-related formin) involved in development and phototaxis was downregulated, which exist in both unicellular and multicellular stages (figure 5F). It was reported that another second messenger c-di-GMP was essential to improve cell differentiation during the multicellular phase. The diguanylate cyclase gene *dgcA* was downregulated under dark incubation, which could also impede the cell differentiation during multicellularity (figure 5F). Overall, we suggested that light as an essential factor that not only induces the phototaxis of amoeba cells but also enhances the cell development and differentiation in both unicellular and multicellular phases.

### 3.5. The metabolomic analysis indicates certain metabolites could influence the migration of amoebae

**Figure 6.**
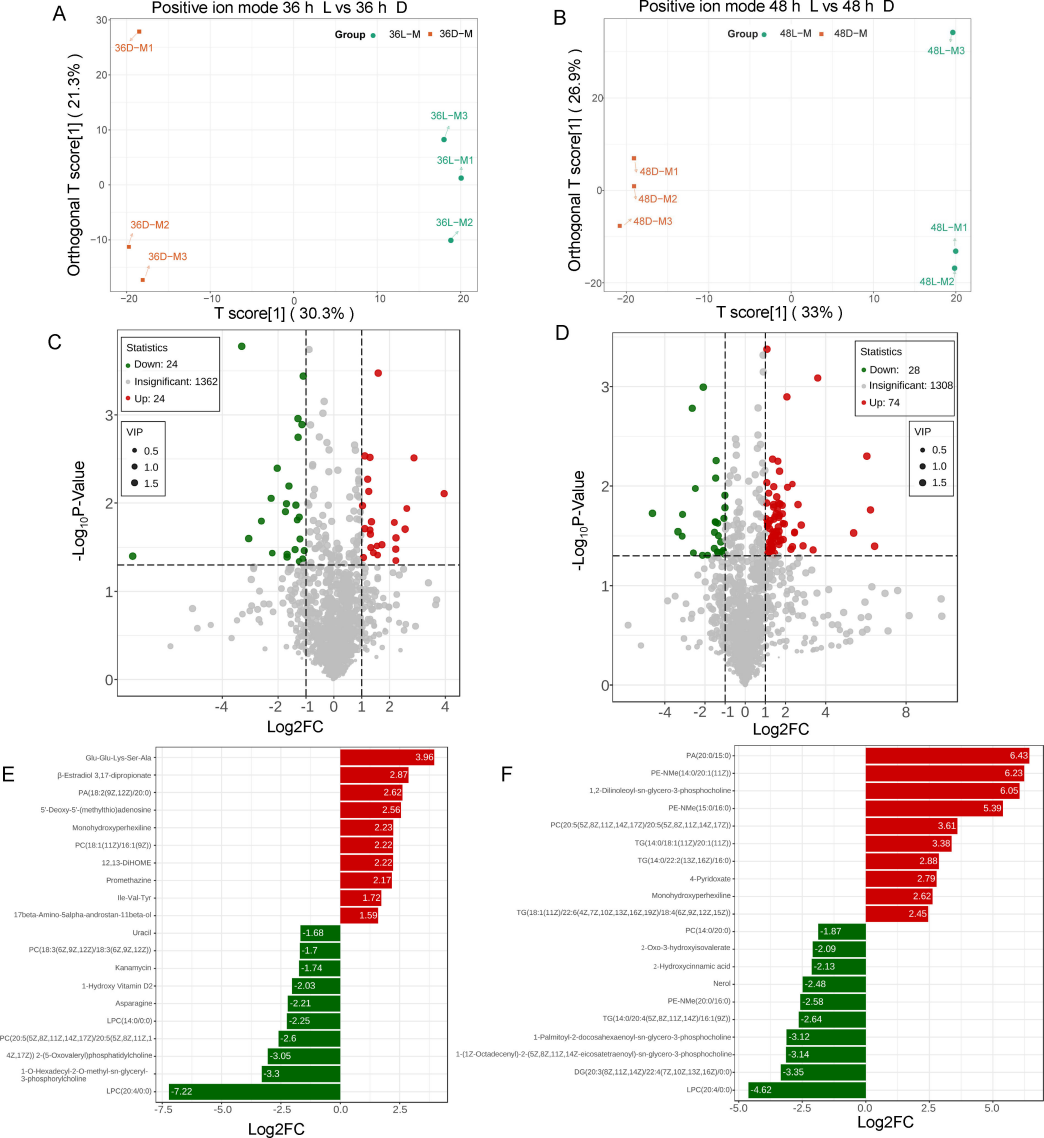
Metabolomic analysis of unicellular and multicellular phase in both dark and light incubations. Three biological replications (amoeba QS9) were conducted using the same batch of cells as described above. Metabolites identified from positive ion mode were presented below. (A,B) OPLS-DA score plots of the DAMs under dark and light treatments in unicellular and multicellular stages, respectively. (C,D) Volcanic plots of DAMs from dark and light incubation in unicellular and multicellular stages, respectively. (E,F) The top 20 significantly metabolic compounds were shown in unicellular and multicellular, respectively.

Meanwhile, we also examined metabolites samples at the unicellular 36 h and multicellular 48 h under both dark and light incubation. The OPLS-DA scoring plot was obtained with an obvious separation of differentially accumulated metabolites (DAMs) between dark and light incubation in both positive ion model (figure 6A,B) and negative ion model (electronic supplementary material, figure S6A,B) for the unicellular and multicellular stages. The *Q*^2^ values ranged from 0.74−0.85, indicating effective models for analysis. Consequently, the OPLS-DA modules were utilized to identify differences in DAMs. DAMs were selected based on the variable importance of the projection (VIP) ≥ 1. |log_2_FC|≥ 1 and *p*-value < 0.05 were designed to extract significantly different metabolites in comparison between dark and light treatment. In the unicellular stage, a total of 48 significant metabolites were identified in positive ion mode, with 24 upregulated and 24 downregulated metabolites under dark treatment (figure 6C). In negative ion mode, fewer significant metabolites were identified, including seven upregulated and four downregulated metabolites (electronic supplementary material, figure S6C). By contrast, more DAMs were identified in the multicellular stage with a total of 102 significant metabolites in positive ion mode—74 upregulated and 28 downregulated metabolites (figure 6D). The negative ion mode showed fewer significant metabolites—13 upregulated and 7 downregulated metabolites (electronic supplementary material, figure S6D). In addition, we selected the top 20 significant metabolites from unicellular and multicellular stages in the positive ion mode (figure 6E,F). The upregulated metabolites were represented by red bars and downregulated metabolites by green bars. Notably, the specific metabolite LPC(20:4/0:0) (lysophosphatidylcholine) was the most significantly downregulated in both unicellular and multicellular phases. Previous studies have reported that LPC can induce the chemotaxis by binding to G protein-coupled receptors, which facilitate the migration of lymphocytes and macrophages. However, it remains uncertain whether the significant downregulation of LPC leads to a reduction in the migration of amoeba cells or slugs. Metabolites were identified from the unicellular (electronic supplementary material, figure S6E) and multicellular (electronic supplementary material, figure S6F) phases in the negative ion mode. Previous studies have reported that GSH (Glutathione) levels are essential for enhancing the multicellular development of *D. discoideum*. Here we observed that GSH (Glutathione) was significantly downregulated in darkness (electronic supplementary material, figure S6F). This may also indicate the reduced GSH (Glutathione) levels under dark could delay multicellularity formation. Furthermore, KEGG pathway enrichment analysis in the unicellular stage indicated that DAMs were mainly enriched in Neomycin, kanamycin and gentamicin biosynthesis (ko00524) linoleic acid metabolism (ko00591), Alanine, aspartate and glutamate metabolism (ko00250), and Pyrimidine metabolism (ko00240) in comparison between dark and light conditions (electronic supplementary material, figure S7A,C). By contrast, DAMs identified in multicellular stage were enriched in metabolism of xenobiotics by cytochrome P450 (ko00980), indole diterpene alkaloid biosynthesis (ko00403), and tropane, piperidine and pyridine alkaloid biosynthesis (ko00960) when comparing dark and light conditions (electronic supplementary material, figure S7B,D). However, the remaining significant metabolites were still not enriched in certain KEGG pathways.

## 4. Discussion

As a well-established model organism, *Dictyostelium discoideum* has been widely used to analyse the transition from unicellular to multicellular development. A variety of environmental cues, including nano- or microplastics and antibiotics, can have effects on the phagocytosis and development of amoebae [31,32]. Copper stress can also modify the dynamic interactions between amoebae and their symbiont bacteria [33]. Symbiont *Paraburkholderia* bacteria in amoebae exhibit increased survival rates under various environmental stresses, such as heat, salt and heavy metal treatments [34]. Notably, light as a persistent environmental factor should be further investigated for its role in regulating amoeba development during the formation of multicellularity. It is already known that the tips of amoeba slugs can respond to light, which determines their migration direction [13]. However, whether the unicellular cells or cell differentiation involved in multicellularity also respond to light remains to be clarified. In this study, we systematically investigated how light exposure can have effects on the amoeba cell development and differentiation as they transition from unicellular to multicellular stages during the life cycle. We observed the different morphologies of amoeba cells under dark and light, and investigated the transcriptomic and metabolomic analysis at the unicellular 36 h stage and multicellular 48 h developmental stage. This study aims to identify the signalling molecules underlying distinct phenotypes observed under light exposure. Our findings will enhance our understanding of the potential conserved mechanisms that facilitate the transition to multicellularity, with light as a key environmental factor.

During the unicellular stage of amoeba cells, it is well known that the cell aggregation can be triggered by starvation. Extracellular cAMP serves as a key chemoattractant signalling molecule that has essential effects on regulating the cell population from quiescence to rhythmic activity [5]. This triggers the chemotaxis of amoeba cells for the aggregation. CarA-1, CarB, CarC and CarD proteins (encoded by genes *carA-1*, *carB*, *carC*, *carD*, respectively) belong to a family of G-protein coupled receptors. They can mediate developmental responses to cAMP, also named cAMP receptors (cARs, cAR1−4) [6]. cAMP receptors play crucial roles in the developmental programme of *D. discoideum* [35], generating pulses of cAMP for chemotaxis from cell aggregation to multicellular mound formation [36]. However, different cARs exhibit distinct expression patterns [37,38]. For example, CAR1 (also named CarA-1, cAR1) is mainly expressed in the early developmental stages. CAR1 is activated by extracellular cAMP and generates pulses of cAMP in the aggregation circuit [39]. CAR2 (CarB) was previously reported to be expressed during post-aggregative development [6]. *car2^−^* cells showed arrest prior to tip formation at the mound stage. This indicates CAR2 is involved in prestalk cell sorting after aggregation [40]. Additionally, cAMP can activate the receptor cAR1 (also named as CarA-1) during both aggregation and post-aggregative development, which is conserved throughout the *Dictyostelia* phylogeny [6,41]. Therefore, the oscillatory cAMP signalling is essential for coordinating aggregation, which was evolved early during *Dictyostelia* evolution [6]. Our study first compared the morphologies of unicellular vegetative of amoeba cells under dark and light incubation conditions. We observed that the vegetative cells exposed to light exhibited more pseudopods compared with those kept in dark treatment (figure 4). This also induced the faster aggregation processes in light than that of under dark. We found that the expression of *acrA* (adenylate cyclase) was downregulated under dark, thus reducing cAMP production and diminishing aggregation among single cells. Previous reports showed that *car2^−^* cells exhibited developmental arrest at the mound stage [40]. In our study, we observed the partial mound arrest under dark condition. Under the same incubation time, tipped mounds or migrating slugs emerged upon exposure to light (electronic supplementary material, figure S8A), while tight aggregation mounds appeared under dark conditions (electronic supplementary material, figure S8B). Furthermore, it is evident that slugs had already formed in the light (electronic supplementary material, figure S8C), whereas tipped mounds exhibit a partial delay under dark conditions (electronic supplementary material, figure S8D). Moreover, the expression of *CarB* was downregulated in dark during the unicellular stage, and this could also explain the delayed cell aggregation and its impact on post-aggregative development. G-box binding factor GbfA and TgrC1 (lagC) have also reported to be involved in the post-aggregation stage [42], both of which were downregulated under the dark condition from transcriptomic analysis in the unicellular phase. Therefore, we suggest that light stimulation enhances the chemotaxis of amoeba cells, promoting more effective aggregation and post-aggregative development.

In the downstream regulatory processes of cAMP, other conserved signalling molecules in *Dictyostelium* also play crucial roles in chemotaxis. For example, Ras GTPases have localization activity triggered by heterotrimeric G proteins at the leading edge of chemotaxing cells [10]. It was reported that RasD is important for cell development and cell-type determination. A Ras pathway can also connect the light stimuli to coordinated cell movement [43]. In multiple Rho small GTPases, Rac1 can regulate actin protrusions, which controls the cell migration [11]. Therefore, multiple small GTPases like Ras and Rac GTPases are involved in the formation of macropinocytic and phagocytic cups, thus regulating the engulfment in *Dictyostelium*. In our study, we observed the shapes of *Dictyostelium* single cells changed more rapidly under light exposure, exhibiting an increase in pseudopodia compared to those under dark incubation (figure 4). Certain Ras and Rho GTPases such as *rasD* and *racL,* etc. were downregulated under dark incubation by the transcriptomic analysis (figure 5F, electronic supplementary material, figure S5). Furthermore, the cell migration is dependent on the actin protrusions. Previous studies have showed that F-actin cross-linking protein filamin (ddFLN) exhibits strong phototaxis in multicellular slugs of *Dictyostelium* [13]. FLN as F-actin binding protein was also named gelation factor or filamin, which involved in actin cytoskeleton organization, motility and development. Here we found that the expression of *FLN* was downregulated under dark, and this may reduce the cell motility and migration during unicellular stage (electronic supplementary material, figure S5A). Overall, this further provides insights that the light exposure can upregulate the core conserved signalling molecules to enhance the cell chemotaxis and promote the cell development and migration during the unicellular phase.

During the multicellular stage, diaphanous-related formins (DRFs) in the downstream of Rho family GTPases can play roles in promoting the formation and elongation of actin filaments [14]. DRF dDia1 was depicted with phototaxis during the multicellular of *Dictyostelium* cells. dDia1-deficient cells impaired the multicellular stage and formed significantly smaller fruiting bodies [14]. Our study observed the development of multicellular slug was impaired and the fruiting bodies became smaller under dark incubation. The gene expression of *drf* (also named *dDia1*) was downregulated under the dark condition (figure 5F). This could also explain that the development of multicellularity was delayed in the dark treatment. As second messenger, c-di-GMP synthesized by diguanylate cyclases normally exists in prokaryote and triggers biofilm formation. DgcA (*Dictyostelium* diguanylate cyclase) can produce c-di-GMP, which can regulate stalk cell differentiation. The DgcA-deficient cells could not form the fruiting bodies [8,9]. Our data determined that the expression of *dgcA* was downregulated under dark. This also proves that the cell differentiation is diminished during the multicellularity in dark. During multicellular development, the *ecmA* and *ecmB* genes encode extracellular matrix proteins, which are mainly expressed in the most anterior prestalk cells of slugs and can regulate the morphogenesis of *Dictyostelium* [44–47]. The anterior prestalk cells are well known to be sensitive to light. Our findings revealed that both of *ecmA* and *ecmB* genes are upregulated when exposed to light (electronic supplementary material, figure S5B). This indicates that the light exposure is involved in establishing multicellular morphogenesis of *Dictyostelium*. As a cellulose-binding domain-containing protein, staA was also engaged in stalk cell differentiation during the multicellular development [48]. We detected that the light illumination can upregulate the expression of *staA*, potentially promoting stalk cell differentiation. Therefore, our data provide additional evidence that light exposure is one of the key factors promoting some conserved signalling molecules during multicellular development. Given the significant impact of light on amoeba development, we also explored whether photoreceptors especially membrane rhodopsins could have effects on the morphogenesis formation of amoeba cells. We found that the expression of *GPR180* (encoded rhodopsin-like GPCR transmembrane domain containing protein) was upregulated in dark incubation. This might give hints that rhodopsin-like GPCR could be accumulated more in dark incubation.

Although a large number of DEGs were identified under dark and light in the unicellular and multicellular stages, the number of significant metabolites of *Dictyostelium* cells identified remains limited in comparison between dark and light treatments. The pigments were previously reported as carotenoids in fruiting body. Our data suggest that these pigments accumulate in the dark and exhibit photo-bleaching effects. Notably, the addition of diphenylamine (DPA) to the culture plates resulted in observable decolourization of the accumulated pigments. This indicates that the pigments likely consist mainly of light-sensitive carotenoids. It was reported that GSH (Glutathione) levels are important to regulate the multicellular development of *D. discoideum*. Without GSH, *Dictyostelium* cells are unable to initiate growth and differentiate into prespore cells during multicellular development [49–51]. In the dark incubation, we clearly observed that the multicellular development was delayed and interrupted to some extent. From the metabolomics analysis, we discovered that glutathione was significantly downregulated under dark treatment and this may also explain the delayed multicellularity formation (electronic supplementary material, figure S6F). Furthermore, Lysophosphatidylcholine (LPC) is mainly contained in the oxidatively damaged low-density lipoprotein (oxLDL), which have many biological functions including cell division, induction of chemotaxis, phagocytosis and the release of oxidative stress, etc. [22]. Under dark incubation, we found that the most significantly downregulated metabolite was determined as an unsaturated arachidonoyl-lysophosphatidylcholine (LPC 20:4) in both unicellular and multicellular stages (figure 6E,F). It is possible that the dark condition may be protecting phosphatidylcholine from being oxidized to become LPC. Whether metabolites like LPC could induce the developmental processes of amoebae needs to be clarified with other investigation strategies such as targeted metabolomics or lipidomics in the future.

In conclusion, this study systematically investigated light exposure as a key environmental factor involved in the regulation of some conserved signalling molecules to promote cell chemotaxis, development, differentiation and multicellular formation during the life cycle of *Dictyostelium* cells. Signalling molecules such as cAMP and c-di-GMP are conserved in the single cell aggregation and multicellular differentiation within the *Dictyostelia* group. Our data suggested that these second messengers could be regulated by light to control the cell development. In the downstream of second messengers, we also observed that multiple small GTPases such as *rasD* and *racL* are upregulated with light exposure, and this provided insights that the cell movement, phagocytosis as well as actin protrusions are coordinated and promoted by light. DRF *dDia1* and *dgcA* are involved in the multicellular development including slugs and fruiting body formation, which can be upregulated by light conditions. Therefore, we propose that light-regulated core signalling pathways could integrate and enhance the transition to the multicellular stage of *Dictyostelium* cells. However, other membrane photosensors and metabolites may also influence the transitions from single cells to multicellular forms, which remain to be investigated in the future.

## Data Availability

Our paper presents new data, which is included in the supplemental files [52].
